# Clinical Features and Maternal-fetal Results of Pregnant Women in COVID-19 Times

**DOI:** 10.1055/s-0041-1729145

**Published:** 2021-06-28

**Authors:** Ana Paula Nogueira Godoi, Gilcelia Correia Santos Bernardes, Leilismara Sousa Nogueira, Patrícia Nessralla Alpoim, Melina de Barros Pinheiro

**Affiliations:** 1 Universidade Federal de São João del-Rei, Divinópolis, MG, Brazil; 2 Universidade Federal de Minas Gerais, Belo Horizonte, MG, Brazil

**Keywords:** SARS-CoV-2, COVID-19, pregnancy, pregnancy complications, high risk pregnancy, SARS-CoV-2, COVID-19, gravidez, complicações na gravidez, gravidez de alto risco

## Abstract

**Objective** Coronavirus disease 2019 (COVID-19) is a disease caused by a newly discovered coronavirus, severe acute respiratory syndrome coronavirus 2 (SARS-CoV-2), which usually leads to non-specific respiratory symptoms. Although pregnant women are considered at risk for respiratory infections by other viruses, such as SARS and Middle East respiratory syndrome (MERS), little is known about their vulnerability to SARS-CoV-2. Therefore, this study aims to identify and present the main studies on the topic, including the postpartum period.

**Methods** In this narrative review, articles were searched in various databases, organizations, and health entities using keywords compatible with medical subject headings (MeSH), such as: *COVID-19* , *pregnancy* , *vertical transmission* , *coronavirus 2019* , and *SARS-CoV-2* .

**Results** The review of the scientific literature on the subject revealed that pregnant women with COVID-19 did not present clinical manifestations significantly different from those of non-pregnant women; however, there are contraindicated therapies. Regarding fetuses, studies were identified that reported that infection by SARS-CoV-2 in pregnant women can cause fetal distress, breathing difficulties and premature birth, but there is no substantial evidence of vertical transmission.

**Conclusion** Due to the lack of adequate information and the limitations of the analyzed studies, it is necessary to provide detailed clinical data on pregnant women infected with SARS-CoV-2 and on the maternal-fetal repercussions caused by this infection. Thus, this review may contribute to expand the knowledge of professionals working in the area as well as to guide more advanced studies on the risk related to pregnant women and their newborns. Meanwhile, monitoring of confirmed or suspected pregnant women with COVID-19 is essential, including in the postpartum period.

## Resumo

**Objetivo** A *Coronavirus disease 2019* (COVID-19) é uma doença causada por um coronavírus recém descoberto, o *severe acute respiratory syndrome coronavirus 2* (SARS-CoV-2), que geralmente leva a sintomas respiratórios não específicos. Embora mulheres grávidas sejam consideradas em risco de infecções respiratórias por outros vírus, como SARS e *Middle East respiratory syndrome* (MERS), pouco se sabe sobre sua vulnerabilidade ao SARS-CoV-2. Portanto, este estudo tem como objetivo identificar e apresentar os principais estudos sobre o tema incluindo o período pós-parto.

**Métodos** Nesta revisão narrativa, foram pesquisados artigos em diversas bases de dados, organizações e entidades de saúde, utilizando palavras-chave compatíveis com o MeSH, tais como: *COVID-19* , *gravidez* , *transmissão vertical* , *coronavírus 2019* , e *SARS-CoV-2* .

**Resultados** A revisão da literatura científica sobre o assunto revelou que as gestantes com COVID-19 não apresentaram manifestações clínicas significativamente diferentes das não gestantes, porém existem terapias contraindicadas. Em relação aos fetos, foram identificados estudos que relataram que a infecção por SARS-CoV-2 em mulheres grávidas pode causar sofrimento fetal, dificuldades respiratórias e parto prematuro, mas não há evidências substanciais de transmissão vertical.

**Conclusão** Devido à falta de informações adequadas e às limitações dos estudos analisados, é necessário fornecer dados clínicos detalhados sobre as gestantes infectadas pelo SARS-CoV-2 e sobre as repercussões materno-fetais causadas por esta infecção. Assim, esta revisão pode contribuir para ampliar o conhecimento dos profissionais que atuam na área, bem como para orientar estudos mais avançados sobre o risco relacionado à gestante e seu recém-nascido. Enquanto isso, o monitoramento de gestantes confirmadas ou suspeitas com COVID-19 é essencial, incluindo o pós-parto.

## Introduction

Coronaviruses (CoVs) are a large viral family, known since the mid-1960s, that cause respiratory infections in humans and animals. Some coronaviruses can cause severe respiratory syndromes, such as severe acute respiratory syndrome (SARS) and Middle East respiratory syndrome (MERS). Severe acute respiratory syndrome is caused by the SARS-associated coronavirus (SARS-CoV), with the first reports being made in China in 2002. Middle East respiratory syndrome, in turn, is a respiratory disease caused by the MERS-CoV. It was identified in 2012, and, since 2016, it has been drastically reduced after public health efforts to prevent transmission of MERS-CoV. 1 2 Recently, a new coronavirus has been identified, SARS-CoV-2, and it is associated with the coronavirus disease 2019 (COVID-19). 1 3 4 5

The most common symptoms at onset of COVID-19 illness are fever, cough, and fatigue, while other symptoms include sputum production, dyspnea, headache, hemoptysis, and diarrhea. 3 6 Some patients with COVID-19 have laboratory changes such as lymphopenia, thrombocytopenia, and elevation of C-reactive protein (CRP). D-dimer elevation can also be identified and serves as an indication of a worse prognosis of COVID-19, although this is already a parameter normally increased in pregnant women. 7 8 Changes in radiographs are common in symptomatic patients with saturation < 95%, manifesting as pneumonia. 7

Many patients can be asymptomatic, which facilitates virus spread. 7 According to the World Health Organization (WHO), on August 10, 2020, there were 19,718,030 people infected globally, with 728,013 confirmed deaths. 9 In Brazil, on the same date, the number of people infected was 3,035,422, with 101,049 deaths, 10 and among these, 199 were puerperal women. 11

Severe acute respiratory syndrome coronavirus and MERS-CoV have caused adverse maternal-fetal outcomes, such as maternal death, intrauterine fetal growth restriction, spontaneous abortion, and premature birth. Thus, considering that these viruses are similar, as they belong to the same genus *Betacoronavirus* , one can admit an adverse potential in pregnant women infected with SARS-Cov-2; 3 4 12 however, due to its recent discovery, little is known about its relationship with pregnancy. Therefore, this review aimed to analyze reports related to SARS-CoV-2 infection in pregnancy and postpartum as well as its consequences in the maternal-fetal sphere in order to assist in the management of these patients.

## Methods

The PubMed, Scopus, Embase, MedRxiv, Science Direct, and Web of Science databases were searched electronically, as well as the websites for national and international health organizations. Only articles published in English and Portuguese were considered. As search strategy, combinations of words related to coronavirus were used, including *severe acute respiratory syndrome* , *SARS* , *vertical transmissio* n, *SARS-CoV-2* , *COVID-19* , and *pregnancy* , until July 29, 2020.

## Results

### Clinical, Laboratory, and Imaging Features in Pregnant Women with Suspected or Proven COVID-19

It is known that pregnant women have a higher risk of severe morbidity and mortality when affected by other respiratory infections, such as influenza and SARS-CoV. Therefore, they should be considered a population at risk for COVID-19. 13 14 15 Adverse maternal-fetal outcomes ( *e. g.* premature birth) have been reported in the literature. However, this information is based on limited data and it is not clear that these results are related to maternal infection. 13

The Brazilian Ministry of Health included high-risk pregnant women in the risk group for complications caused by SARS-CoV-2 infection. It also emphasizes that urgent measures for specific clinical management must be respected for this population, such as early medication and not delaying radiographic exams regardless of the gestational period. 15 16 In addition, the possibility of worsening the infection caused by SARS-CoV-2 in pregnant women cannot be ruled out. 17 The coronavirus clinical management protocol (COVID-19) in primary health care of the Brazilian Ministry of Health has also emphasized the relocation of health professionals who are pregnant, especially if their pregnancy is high-risk. Furthermore, this protocol also establishes that both pregnant and puerperal women should receive priority care. 15

Therefore, pregnant women with SARS-CoV-2 infection, even with a mild course, should be monitored including bi-monthly fetal growth ultrasound monitoring and Doppler assessment, due to the potential risk of restricted fetal intrauterine growth. 18 Due to the delay in reverse transcript polymerase chain reaction (RT-PCR) tests, chest computed tomography (CT) in the third trimester may be an effective way to screen for COVID-19 pneumonia in pregnant women, particularly in areas with outbreaks in progress. 19

In a study by Ellington et al., 20 data were collected from 91,412 women diagnosed with COVID-19, aged 15 to 44 years, 8.98% of whom were pregnant. Symptoms were reported by 97.7% of pregnant women and 96.2% of non-pregnant women. However, the risk of hospitalization was 5.4 times higher for pregnant women, while the risk of admission to the intensive care unit (ICU) and mechanical ventilation was 1.5 and 1.7, respectively, compared to the group of non-pregnant women. 20 In addition to the common laboratory findings in people with COVID-19, all pregnant women with SARS-CoV-2 pneumonia also presented D-dimer levels above the normal range, even considering the normal elevation usually found in pregnancy. Two (29%) patients had different degrees of abnormal liver function, as well as an increase in alanine aminotransferase (ALT) and/or aspartate aminotransferase (AST). Interleukin-6 was tested in four patients, all with levels above the normal range. Two patients had chronic diseases (polycystic ovaries and hypothyroidism) and three had co-infection (two due to H1N1 and one due to *Legionella pneumophila* ). According to chest computed tomography (CT), 6 (86%) patients had bilateral pneumonia, and the rest (14%) had unilateral pneumonia. After the follow-up period, all patients were discharged from the hospital. Four neonates were released without testing for SARS-CoV-2, and there were no signs of fever or pathological jaundice after 28 days. Three neonates were under observation and were tested, the result was positive in 1 of them 36 hours after birth, even with negative viral tests of cord blood and placenta. The neonate with a positive test did not have a fever or cough, had mild signs of breathing difficulty and a chest X-ray revealed mild pneumonia. After 28 days of life, the baby had two negative results on the molecular test and was discharged. 8

Wu et al. 21 evaluated 23 pregnant women with COVID-19, most of whom were asymptomatic (n = 15). Among the asymptomatic pregnant women, six were at risk of miscarriage or premature rupture of the membrane. When comparing the average hospital stay, asymptomatic patients had a shorter hospital stay (14 days) than symptomatic patients (25.5 days). 21

Physiological gestational changes and pathological disorders, such as endocrine and/or vascular disorders, which occur during high-risk pregnancies, may influence the pathogenesis and/or clinical presentation of SARS-CoV-2 infection in pregnant women. 22 The human placenta expresses an excessive amount of the angiotensin-converting enzyme 2 (ECA2), 23 which is the SARS-CoV-2 cell receptor, 24 whose main function is to regulate blood pressure and fetal development. 23 Thus, a possible intrauterine infection by COVID-19 can alter the ACE2 expression and trigger hypertensive complications during pregnancy, such as preeclampsia. 22

**Fig. 1:**
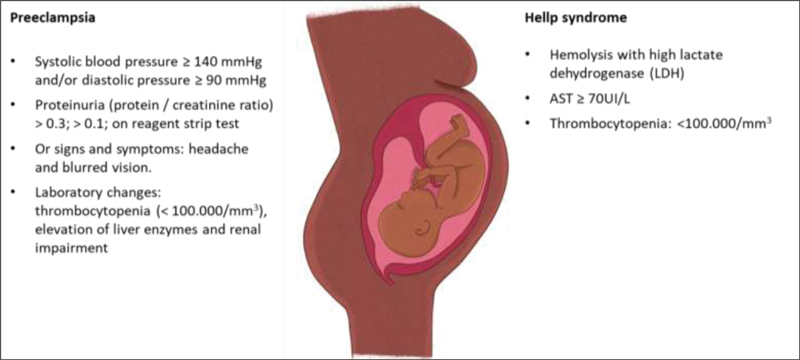
Most frequent complications in pregnancy.

Hypertensive syndromes are the most frequent complications in pregnancy and are the leading cause of maternal death in Brazil, mainly in its severe forms, such as preeclampsia and hemolysis, elevated liver enzymes, low platelet count (HELLP) syndrome. 25 Figure 1 shows the most frequent complications in pregnancy.

In a study by Mendoza et al., 26 42 pregnant women with gestational age greater than 20 weeks and diagnosed with COVID-19 were classified as severe and non-severe according to the type of pneumonia. Eight pregnant women developed severe pneumonia requiring admission to the intensive care unit (ICU), and 6 of these women had characteristics of preeclampsia. When analyzing the criteria for preeclampsia/HELLP syndrome, it was found that only one case had all the requirements (increased lactate dehydrogenase [LDH], placental subperfusion, and abnormal angiogenic state). Thus, the authors suggested that the other 5 cases of preeclampsia can be explained by complications related to COVID-19. 26

Although there are few specific data on SARS-CoV-2, other analyzed viruses and respiratory viruses can bring serious conditions to pregnant women and should guide the care of pregnant women with COVID-19 until additional data becomes available. Given the above, the consensus among experts was that pregnant women should be isolated to avoid contamination. 1

According to Zhang et al. (2020), 27 even the infection with mild symptoms of COVID-19 reduces lung function. Therefore, with early isolation and drug treatment, cases are less likely to progress to severe pneumonia. However, vigilance should be increased, and, if necessary, pregnancy must be interrupted as soon as possible to prevent the development of the disease to severe and critical stages. At the same time, multidisciplinary cooperation is essential to jointly guarantee the safety of the mother-child binomial. 27

Even during the pandemic, research is conducted on the impacts of COVID-19 infection on the clinical presentation and perinatal and/or puerperal outcomes; however, the data are still limited and are not conclusive regarding the risk of developing severe forms of COVID-19 associated with pregnancy. However, due to the physiological changes of the gestational period, pregnant women can be seriously affected by some infections. Therefore, it is important to adopt precautionary measures against COVID-19 and systematic monitoring of pregnant women, even if this monitoring occurs in the non-face-to-face care. Table 1 shows the findings of the main studies involving pregnant women with COVID-19 and their newborns.

**Table 1:** Main results published on pregnant women with COVID-19 and their newborns

Number of pregnant patients	Delivery route	Maternal symptoms	Maternal/fetal complications	Study	Date of publication
09	Cesarean section	Seven patients had fever, four had cough, and two had malaise	One had flu, one had gestational hypertension, one had pre-eclampsia, two had fetal distress, and three had a ruptured membrane	(Chen et al., 2020) 28	March 07, 2020
17	Cesarean section	Four had fever, four had cough, one had fatigue, two had chest pain, one had dyspnea, and one had diarrhea	Three underwent emergency cesarean section	(Chen et al., 2020) 29	March 16, 2020
13	Ten cesarean sections, five of which were emergency. Three pregnant women were still pregnant at the end of the study	Ten had fever, three had dyspnea, and one was asymptomatic	Three had fetal distress, one had a ruptured membrane, and one was stillborn	(Liu et al., 2020) 30	March 5, 2020
01	Spontaneous vaginal	Fever, cough, headache, and myalgia	Gestational hypertension and hypothyroidism.	(Zambrano et al., 2020) 31	March 25, 2020
07	Seven cesarean sections, two of which were emergency due to preeclampsia	Six had fever, one had cough, one had shortness of breath, and one had diarrhea.	Two patients had hypertension, blurred vision, and preeclampsia.	(Yang et al., 2020) 32	June 2020
01	Vaginal delivery	Fever and dry cough	Premature rupture of the membrane	(Xiong et al., 2020) 33	April 10, 2020
01	Emergency cesarean section	Fever	Intermittent fever in the postoperative period.	(Wang et al., 2020) 34	March 12, 2020
23	Eighteen cesarean sections, two vaginal deliveries and three patients voluntarily terminated the pregnancy in the first trimester	Four patients had fever, six had cough, one had nasal congestion, and 15 patients were asymptomatic	One had fetal intrauterine hypoxia, two had a ruptured membrane, four had gestational hypertension, and three had threat of miscarriage	(Wu et al., 2020) 21	April 8, 2020
03	Vaginal deliveries	Fever, cough, and chest tightness	No complications	(Khan et al., 2020) 35	March 19, 2020
04	Three cesarean sections and one vaginal delivery	Three had fever, two had cough, two had myalgia/fatigue, and two had headache	Two newborns had edema and rash	(Chen et al., 2020) 36	March 16, 2020
07	Cesarean	Six patients had fever, one had cough, one had shortness of breath, and one had diarrhea	Three patients had uterine scars	(Yu et al., 2020) 8	March 24, 2020
15	Ten cesarean sections, a vaginal delivery, and four patients were still pregnant at the end of the study	Thirteen had fever, nine dyspnea, three myalgia, one diarrhea, one cough and one fatigue	Mild clinical manifestations	(Liu et al., 2020) 37	July 2020
01	Emergency cesarean section	Fever	No complications	(Wang et al., 2020) 38	February 28, 2020
09	Seven cesarean sections and two vaginal deliveries	Eight patients had fever, four had cough, one had diarrhea, and one had sore throat	Five neonates were cured, four remained in the hospital until the end of the study, and one died	(Zhu et al., 2020) 39	February 09, 2020
01	Vaginal delivery	Fever, chills, dry cough, and myalgia	No complications	(Iqbal et al., 2020) 40	April 01, 2020
05	Three vaginal deliveries, one cesarean section due to gestational diabetes and one emergency cesarean section due to fetal tachycardia	All pregnant women had postpartum fever	Two patients developed gestational diabetes, and one developed preeclampsia	(Chen et al., 2020) 41	March 28, 2020
01	Cesarean section	Fever, nasal congestion, and respiratory distress	No complications	(Dong et al., 2020) 42	March 26, 2020
02	Cesarean section	Two patients had fever, two had nasal congestion, and one had chills. One had fever, nasal congestion, sore throat, and a rash	No complications	(Fan et al., 2020) 43	March 17, 2020
Case: 16 COVID-19 pregnant women Control: 45 healthy pregnant women	Caesarean section	Fifteen pregnant women with COVID-19 had mild pneumonia, and one of them had severe pneumonia	One patient in the control group was in a more serious condition. No patient in the case group was in critical condition	(Zhang et al., 2020) 27	March 25, 2020
8.207	Not informed	1,799 patients had cough, 1,190 had fever, 1,323 had myalgia, 989 chills, 1,409 had headache, 497 had diarrhea, 682 had nausea or vomiting, 350 had abdominal pain, 326 had runny nose, and 587 had new loss of taste or smell	Risk of hospitalization 5.4 times higher than non-pregnant women. Risk of being admitted to the ICU and receiving mechanical ventilation was 1.5 and 1.7, respectively, compared to the group of non-pregnant women	(Ellington et al., 2020) 20	June 26, 2020

Abbreviations: COVID-19, coronavirus disease 2019; ICU, intensive care unit.

### Management of Pregnant Women with COVID-19

Prenatal and postpartum care cannot be postponed or canceled. Therefore, maternity services must be adapted quickly to provide safe care, minimizing the risk of spreading COVID-19. Unfortunately, health services will suffer from lack of professionals, as they also become ill and/or need to isolate themselves during this pandemic period. 44 45

According to the Brazilian Ministry of Health (2020), 46 prenatal consultations should take place in a timely manner for pregnant women who do not have flu-like symptoms, paying attention to the prevention of agglomerations and the best hygiene practices. Pregnant women with flu-like symptoms, on the other hand, must have their elective procedures (consultations and routine exams) postponed for 14 days and, when necessary, be seen in an isolated place from other patients. 46 However, it is worth mentioning that depending on the region of the country, there may be specific guidelines. As an example, in Minas Gerais (Brazil), the State Department of Health stated in a technical note issued on April 1, 2020 that in an area with a high flow due to the COVID-19 pandemic, the flexibility of prenatal consultations at usual risk may occur, at clinical criteria. However, the minimum number of consultations and examinations recommended by the Ministry of Health of Brazil and the World Health Organization must be maintained. 47

In a randomized, double-blind study conducted in the United States and Canada, the use of hydroxychloroquine as postexposure prophylaxis was evaluated. The participants were divided into two groups, 414 received hydroxychloroquine and 407 received placebo. All participants had home or occupational exposure to patients diagnosed with COVID-19. The results of this study demonstrated that the use of hydroxychloroquine as postexposure prophylaxis has no benefits. 48

A retrospective cohort study was carried out with 1,438 patients admitted to 25 hospitals in New York to assess the association between hospital mortality caused by SARS-CoV-2 and the use of hydroxychloroquine or azithromycin. The authors concluded that there were no statistically significant differences in mortality between groups. 49

It is very important to carry out an adequate clinical evaluation, establish criteria and prioritize the use of drugs indicated by the WHO (through the Solidarity study) and the Brazilian Ministry of Health, even if there is still no specific treatment for COVID-19. Other drugs should be used in very severe cases and in the absence of response to therapies. 50

It is worth mentioning that, for some drugs, there are already more robust reports, but still without solid evidence of use in critically ill patients and not in mild cases. Therefore, caution, equilibrium, and common sense, combined with controlled scientific studies should be used to deal with this pandemic therapeutically. Tables 2 and 3 shows the therapies under study against COVID-19 infection.

**Table 2:** Drugs recommended by the World Health Organization, in the Solidarity study, for the treatment of COVID-19, on July 6, 2020

Drug	Use in pregnant women	References
Remdesivir	It was not possible to evaluate the effectiveness and/or safety of its use in pregnant women. The Food and Drug Administration (FDA) has authorized its compassionate use for the treatment of severe COVID-19 in children and pregnant women.	(McCoy et al., 2020) 51 (Lim et al., 2020) 52
Lopinavir/ritonavir with interferon beta-1	It presented a good safety profile in its use in pregnant women. The results of this study with Interferon beta 1 suggested that there was no increased risk of miscarriage or congenital anomalies.	(Tookey et al., 2016) 53 (Hellwig et al., 2020) 54

**Table 3:** Drugs recommended by Brazilian Ministry of Health for the treatment of COVID-19

Drug	Use in pregnant women	References
Chloroquine	Chloroquine can induce adverse ophthalmic and cardiac effects on the fetus. It is also genotoxic. Use should be carefully assessed.	(Lacroix et al., 2020) 55
Hydroxychloroquine	In the treatment of autoimmune diseases, it is not associated with any increased risk of birth defects, spontaneous abortions, fetal death, or prematurity. Hydroxychloroquine can induce adverse ophthalmic and cardiac effects on the fetus. It is also genotoxic. Use should be carefully assessed.	(Lacroix et al., 2020; Sperber et al., 2009) 55 56
Azithromycin	In most studies, there were no significant associations between the use of azithromycin in pregnant women and congenital malformations.	(Keskin-Arslan et al., 2020) 57

### Maternal-fetal Care

According to the United States Center for Disease Control and Prevention (CDC), health professionals should follow some recommendations when performing obstetric procedures in pregnant patients with confirmed or suspected COVID-19 diagnosis, from prehospitalization to discharge of the mother and baby. 51 52 53 54 55 56 57 58 59 60 It is worth mentioning that, in Brazil, the diagnosis in pregnant women must follow the same protocol for the general adult population and that attention should be paid to the signs and symptoms that demonstrate clinical severity. 46

Prehospital care includes notification of the obstetrics unit for the proper delivery room preparation, for the correct use of personal protective equipment (PPE) by the health professionals involved and conduct in accordance with biosafety rules. 46

During hospitalization, care must be taken to avoid new infections, and newborn isolation should be discussed with health professionals. If the mother expresses the desire to breastfeed, she should be instructed on the precautions to be followed, such as proper hygiene, use of the breast pump or use of a mask, if she chooses to breastfeed. 46 The benefits of breastfeeding outweigh any potential risks of transmitting the virus through breast milk. 25 61

The American College of Obstetricians and Gynecologists recommends that doctors should follow the CDC's Interim Clinical Guidelines for the management of patients with confirmed coronavirus disease (COVID-19). 13 Recommendations are that even if the ideal maternal-newborn care plan is to maintain this binomial, the temporary separation of the newborn from a mother with confirmed or suspected COVID-19 should be strongly considered to reduce the risk of transmission to the newborn. 62

The WHO recommends that the mother and the newborn should stay together and practice skin-to-skin contact, including hygiene and respiratory care for the mother, especially immediately after birth and during breastfeeding establishment, if the mother or her babies are cases suspected or confirmed of COVID-19. 63

Delivery routes should be individualized based on the obstetric indications and preferences of the pregnant woman. Cesarean section is ideally performed only when clinically justified. Decisions on the corticosteroids use for fetal pulmonary maturation, emergency childbirth, and termination of pregnancy are challenging conditions and must be based on many factors, such as gestational age, severity of maternal condition, and fetal viability and well-being, within a multiprofessional assessment. 64 65

### Vertical Transmission of SARS-Cov-2

A systematic review article 66 included 24 studies that analyzed the effects of COVID-19 on pregnant women and newborns. Regarding clinical symptoms, fever was the most common symptom, occurring in 62.9% of patients, coughing in 36.8%, and sore throat in 22.6%. All deliveries were carried out in a negative pressure room, and care was taken to avoid contamination of the 94 newborns, 31 of whom were premature. The average birth weight was 3,127.6g. Two neonates tested positive for COVID-19. Amniotic fluid, placental fluid, umbilical cord, and gastric juice tested negative. There were three fetal deaths, two due to multiple organ failure and disseminated intravascular coagulation, and the other death because the neonate was cyanotic. No case of severe neonatal asphyxia was observed.

The placenta and decidua are the main interfaces between the mother and the fetus during pregnancy. 67 And, as already reported, the human placenta expresses ACE2 23 and, therefore, may be fundamental for the vertical transmission of SARS-CoV-2. 22 However, the COVID-19 impact on the intrauterine environment is still unclear, as well as whether vertical transmission occurs during a maternal infection. The main studies that have assessed the possibility of vertical transmission are described in Table 4 .

**Table 4:** Main studies assessing the possibility of vertical transmission of SARS-CoV-2

Number of pregnant women	Age of pregnant women	Pregnancy period	Premature birth	Average birth weight	1-minute Apgar score	5-minute Apgar score	Vertical transmission signals	Study	Date of publication
1	30 years	35 weeks	One newborn	Not informed	Not informed	Not informed	RT-PCR not detected	(Li et al., 2020) 68	26 de junho de 2020
9	26–40 years	36–39 weeks + 4 days	4 newborns	Two newborns had low birth weight	8–9	9–10	Six newborns had RT-PCR undetected at birth. It was not possible to investigate the other newborns at the time of birth.	(Chen et al., 2020) 28	March 07, 2020
17	28.7–29.5 years	Three pregnant women: < 37 weeks 14 pregnant women: ≥ 37 weeks	Three newborns	Not informed	7–9	9–10	RT-PCR not detected	(Chen et al., 2020) 29	March 16, 2020
13	22–36 years	25–38 weeks	6 newborns	Not informed	Nine newborns had an Apgar score of 10	Not informed	RT-PCR not detected	(Liu et al., 2020) 30	March 05, 2020
01	41 years	31 weeks	One newborn	1,500 g	Not informed	Not informed	RT-PCR not detected in nasopharyngeal sample	(Zambrano et al., 2020) 31	March 25, 2020
07	Not informed	36–37 weeks	Four late preterm infants	2,096 g ± 660 g	8–9	9–10	RT-PCR not detected	(Yang et al., 2020) 32	June 2020
01	25 years	38 weeks + 4 days	No	3,070 g	9	10	RT-PCR not detected in samples of amniotic fluid, smear of the newborn's throat and rectum.	(Xiong et al., 2020) 33	April 10, 2020
01	34 years	40 weeks	No	3,205 g	8	9	RT-PCR detected from pharynx swab collected 36 hours after birth. Cord and placenta samples were negative.	(Wang et al., 2020) 34	March 12, 2020
23	21–37 years	Twenty pregnant women: > 28 weeks 3 pregnant women: < 12 weeks.	Not informed	Not informed	Not informed	9–10	RT-PCR not detected in 04 newborns. The SARS-CoV-2 infection was ruled out in the others using diagnostic criteria.	(Wu et al., 2020) 21	April 08, 2020
03	27–33 years	34 weeks + 6 days 39 weeks + 1 day 38 weeks + 2 days.	One newborn	3,373 g	8–9	9–10	RT-PCR not detected	(Khan et al., 2020) 35	March 19, 2020
04	28–34 years	37 weeks + 2 days 39 weeks 37 weeks + 3 days 38 weeks + 4 days	None	3,400 g	7–8	8–9	RT-PCR not detected in three newborns. The other parents did not authorize.	(Chen et al., 2020) 36	March 16, 2020
07	29–34 years	37–41 weeks	None	3,264 g	8–9	9–10	Four newborns have not been tested. Of the three who were tested, one tested positive 36 hours after birth	(Yu et al., 2020) 8	March 24, 2020
15	23–40 years	12–38 weeks	None	Not informed	8	9	RT-PCR not detected	(Liu et al., 2020) 37	July 2020
01	28 years	30 weeks	One newborn	1,830 g	9	10	RT-PCR not detected	(Wang et al., 2020) 38	February 28, 2020
09	average age 30 years	31–39 weeks	Six newborns	2,423 g	7–10	8–10	RT-PCR not detected	(Zhu et al., 2020) 39	February 09, 2020
01	34 years	39 weeks	None	Not informed	8	9	RT-PCR not detected	(Iqbal et al., 2020) 40	April 1, 2020
05	25 to years	38 to 41 weeks	None	3,691 g	10	10	RT-PCR not detected	(Chen et al., 2020) 41	March 28, 2020
01	29 years	34 weeks + two days	Not informed	3,120 g	9	10	RT-PCR not detected, High IgM and IgG	(Dong et al., 2020) 42	March 26, 2020
02	29 and 34 years	36 weeks + five days 37 weeks	Not informed	3,145 g	9	10	RT-PCR not detected	(Fan et al., 2020) 43	March 17, 2020
06	Not informed	Not informed	Not informed	Not informed	8–9	9–10	RT-PCR not detected. In two newborns high levels of IgM and IgG. And in three newborns normal IgM and elevated IgG.	(Zeng et al., 2020) 69	March 26, 2020
Case: 16 COVID-19 pregnant women Control: 45 healthy pregnant women	Case: 24–34 years Control: 24–40 years	Not informed	One newborn from the case group	Case: 2,300–3,750 g Control: 2,180–4,100g	Not informed	Not informed	RT-PCR not detected in 10 newborns.	(Zhang et al., 2020) 27	March 25, 2020

Abbreviations: COVID-19, coronavirus disease 2019; IgG, immunoglobulin G; IgM, immunoglobulin M; RT-PCR, reverse transcription polymerase chain reaction; SARS-CoV2, severe acute respiratory syndrome coronavirus 2.

The data to assess the COVID-19 severity in pregnant women are scarce, since most studies had a limited number of participants. It is important to keep in mind that the ideal is to do everything possible to minimize the chance of these patients contracting disease, and, if they do, the measures recommended by the Brazilian Ministry of Health and WHO should be adopted immediately.

## Concluding Remarks

As previously mentioned, studies evaluating the consequences of COVID-19 in pregnant women are scarce and have a limited number of participants, which often generate inconclusive data. Clinical manifestations in pregnant women are similar to those of non-pregnant patients, and there is still no scientific evidence of vertical transmission of SARS-CoV-2. When confirming or suspecting COVID-19 infection in pregnant women, professional follow-up is essential, and all precautions should be taken to minimize the impacts of the disease. Based on the clinical consequences due to the occurrence of pneumonia of other etiologies during pregnancy, there is a theoretical risk of COVID-19 determining unfavorable fetal repercussions. It is necessary that data on pregnant women infected with SARS-CoV-2 as well as its maternal-fetal repercussions are carefully and thoroughly analyzed and made available during the pandemic. Therefore, more detailed studies and specially designed to assess the effects of COVID-19 on pregnant women and their newborns are mandatory to fill this gap that still exists.
